# Optimal multistage designs for randomised clinical trials with continuous outcomes

**DOI:** 10.1002/sim.4421

**Published:** 2011-12-05

**Authors:** James MS Wason, Adrian P Mander, Simon G Thompson

**Affiliations:** a Hub for Trials Methodology Research, MRC Biostatistics UnitCambridge, UK; b MRC Biostatistics UnitCambridge, UK

**Keywords:** phase II clinical trial, optimal design, simulated annealing, admissible design

## Abstract

Multistage designs allow considerable reductions in the expected sample size of a trial. When stopping for futility or efficacy is allowed at each stage, the expected sample size under different possible true treatment effects (*δ*) is of interest. The *δ*-minimax design is the one for which the maximum expected sample size is minimised amongst all designs that meet the types I and II error constraints. Previous work has compared a two-stage *δ*-minimax design with other optimal two-stage designs. Applying the *δ*-minimax design to designs with more than two stages was not previously considered because of computational issues. In this paper, we identify the *δ*-minimax designs with more than two stages through use of a novel application of simulated annealing. We compare them with other optimal multistage designs and the triangular design. We show that, as for two-stage designs, the *δ*-minimax design has good expected sample size properties across a broad range of treatment effects but generally has a higher maximum sample size. To overcome this drawback, we use the concept of admissible designs to find trials which balance the maximum expected sample size and maximum sample size. We show that such designs have good expected sample size properties and a reasonable maximum sample size and, thus, are very appealing for use in clinical trials. Copyright © 2011 John Wiley & Sons, Ltd.

## 1. Introduction

When investigating a novel treatment in a randomised clinical trial, there are substantial advantages to be gained from using a multistage design. At each interim analysis, one may allow the option to stop the trial for futility, when the results so far show that the treatment is unlikely to be judged to be effective, or for efficacy, when the results so far provide sufficient evidence to reject the null hypothesis of no treatment advantage.

Group-sequential designs are multistage designs in which equal numbers of patients are recruited at each stage. If interim analyses are used within a frequentist paradigm, the significance level at each interim analysis must be smaller than the overall significance level required. The Pocock design [1] uses the same thresholds throughout, whereas O'Brien and Flemming's design [2] has stricter thresholds at the initial stages and more liberal ones at later stages.

The purpose of a multistage design is to reduce the expected sample size, but this depends on the true treatment effect. A larger observed treatment effect will mean the trial is more likely to stop early for efficacy but less likely to stop early for futility. An optimal design is one which has the minimum expected sample size conditional on a specific value for the true treatment effect.

Designs that are optimal conditional on a specific treatment effect are not necessarily good choices in practice. This is because the true treatment effect, *δ*, may deviate from the designed value and the performance of the design may be poor. Shuster [3] proposed a design, which minimises the maximum expected sample size for a one-arm trial with binary treatment responses. Wason and Mander [4] explored this design for two-stage two-arm trials with continuous treatment responses; the design was referred to as the *δ*-minimax design. Both previous papers show that the *δ*-minimax design performs well in terms of expected sample size for a wide range of possible treatment effects. On the other hand, the maximum sample size tended to be higher.

In this paper, we extend the *δ*-minimax design to trials with more than two stages. We compare the *δ*-minimax design with other optimal designs for a range of true treatment effects. We also compare it with the triangular design proposed by Whitehead and Stratton [5], which has been shown to minimise the maximum expected sample size as the type I error converges to 0 for designs where the types I and II error probabilities are the same and where there is an interim analysis after a patient from each arm is observed [6]. Trials with these properties are called symmetric and sequential, respectively. We investigate the similarity between the *δ*-minimax design and the triangular design for group-sequential nonsymmetric designs. We then address the issue of high maximum sample sizes by extending the admissible designs of Jung *et al.* [7] to designs that balance the maximum expected sample size and the maximum sample size.

## 2. Methods

### 2.1. Multistage designs

We consider a randomised two-arm group-sequential multistage design with *K* analyses. Patients are recruited in groups of fixed size, with the *i*th analysis taking place after *n*_*i*_ = *in*_1_ patients have been randomised to each arm, and their treatment response measured. We assume that the outcomes are normally distributed with variance *σ*^2^. Defining *δ* as the mean difference in response between the treatment arm and control arm, the hypothesis being tested is H _0_ : *δ ≤ δ*_0_ with the probability of rejecting the null being *≤ α* under H _0_ and *≥*1 −*β* when *δ ≥ δ*_1_, where *δ*_1_ is the clinically relevant difference (CRD). For now, we assume the variance is known. Later, we discuss and assess two straightforward procedures to control the type I error when the variance differs from the presumed value.

At interim analysis *i*, the *z*-statistic is calculated as follows:


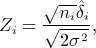
(1)

where 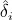 is the maximum likelihood estimator of the treatment effect up to stage *i*. If *Z*_*i*_ > *e*_*i*_, the trial is stopped for efficacy; if *Z*_*i*_ *≤ f*_*i*_, the trial is stopped for futility. If it is between the two thresholds, the trial continues to stage *i*+ 1. Some designs allow the final stage to be inconclusive 8,9, but we consider a design that has *e*_*K*_ = *f*_*K*_ so that a decision one way or the other is made.

For a given design, parameterised by (*n*_1_,*f*_1_, …, *f*_*K*−1_,*e*_1_, …, *e*_*K*_), which continues to the end, a sequence of *z*-statistics will be found, *Z*_1_, …, *Z*_*K*_. The vector of the *z*-statistics, *Z* = (*Z*_1_, …, *Z*_*K*_), is multivariate normal, with *Z*_*i*_ having mean 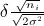 and variance 1. The covariance between *Z*_*i*_ and *Z*_*j*_, with *i* < *j*, is 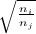 [10]. To find the probability of rejecting H _0_ when the treatment effect is *δ*, we sum the probabilities of rejecting *H*_0_ at each stage:


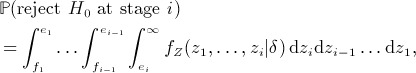
(2)

where *f*_*Z*_ is the PDF of the multivariate normal distribution with mean vector and covariance matrix as previously described.

There are different methods to evaluate the multidimensional integral in Equation (2). One directly integrates the multivariate normal density and is described in Genz and Bretz [11]. The second method uses the fact that (*Z*_1_, …, *Z*_*K*_) is a Markov sequence and is described in Jennison and Turnbull [10]. We have implemented both methods in C + + and have found that the former is quicker computationally when the number of stages is 2 or 3, whereas the second is quicker when the number of stages is higher.

Evaluating Equation (2) for *δ* = *δ*_0_ gives the significance level of the design, with its value under *δ* = *δ*_1_ giving the power. A design for which the significance level and power meet the required constraints is called feasible.

### 2.2. Expected sample size and worst-case scenario treatment effect

For a given group-sequential design, *d*, with parameters (*n*_1_,*f*_1_, …, *f*_*K*−1_,*e*_1_, …, *e*_*K*_), the expected total sample size per arm (*N*) is a function of the probabilities of stopping at each interim analysis and *n*_1_:


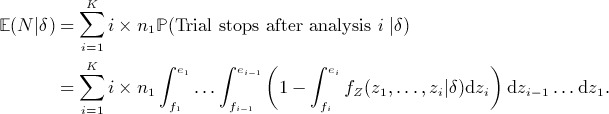
(3)

The expected sample size is a function of *δ*, monotonically increasing to a maximum, and then monotonically decreasing. To see why this is the case, we can rewrite the expected sample size as follows:


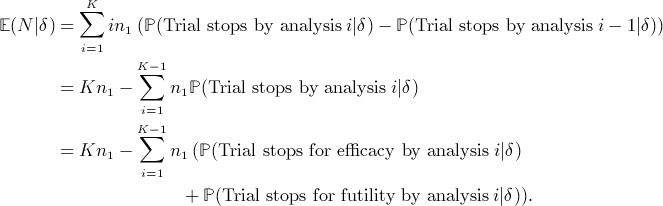
(4)

Then, using the fact that probability of stopping for efficacy by a given analysis increases monotonically with *δ* and the probability of stopping for futility decreases monotonically, this shows that equation (4) behaves as stated earlier and, specifically, has a unique maximum.

To find the value of *δ*, 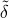, which gives the highest expected sample size for a particular design, we perform a simple interval search. Accuracy to the second decimal point of expected sample size requires around 10 function evaluations.

### 2.3. Optimal designs

An optimal design is one that is feasible and has the lowest expected sample size at a particular treatment effect, *δ*. We restrict our attention to three designs. The first is the null-optimal design, which is optimal for the null treatment effect *δ* = *δ*_0_. The second is the CRD-optimal design, optimal at the CRD *δ* = *δ*_1_. The third is the feasible design with the lowest maximum expected sample size, which we call the *δ*-minimax design. The worst-case scenario treatment effect, 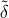, differs depending on the design.

If 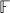 is the set of feasible designs, then the *δ*-minimax design can be defined as follows:


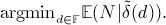
(5)

### 2.4. Dynamic programming method to find null-optimal and CRD-optimal designs

In two-stage designs, it is computationally possible to search over all parameters using a grid search 4, but for each extra stage, the number of parameters is increased by two (a new futility and efficacy threshold). The time to integrate the multivariate distributions in Equations (2) and (3) also increases. Thus, grid searches become impractical when the number of stages is above 2.

As an alternative, the method of dynamic programming has been suggested 12,13,14. The method for nonsymmetric (i.e. *α* ≠ *β*) group-sequential designs is described in Barber and Jennison [15] and is the technique we use here to find the null-optimal and CRD-optimal designs.

### 2.5. Using simulated annealing to find *δ*-minimaxdesigns

It is not obvious how the method of dynamic programming could be applied to find the *δ*-minimax design. This is because the dynamic programming techniques assumes that the value of *δ* for which the design is being optimised does not change. The value of 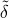 changes as the design parameters do, and so a different method must be used.

We propose the use of simulated annealing, a stochastic search technique described in Lin and Geyer [16]. The function we are interested in minimising is the maximum expected sample size, subject to the design being feasible. To include these constraints, the function to be minimised is the sum of the maximum expected sample size and a penalty factor given to nonfeasible designs. It is desirable that designs that are almost feasible are penalised less than the ones that are far from feasible. If the actual types I and II error probabilities of a design are *α*′ and *β*′, with *α* and *β* the required types I and II error probabilities, then a possible penalty function is as follows:


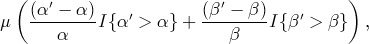
(6)

where *μ* is a fixed penalty factor and *I*{}is the indicator function. As we discuss later, a higher value of *μ* will discourage the process from moving to designs that are not feasible. This would be undesirable if the process is stuck in a region with a local optimum. If it is too low however, the process may get stuck in a region where all designs are nonfeasible. We set *μ* to be the value of the sample size for the one-stage design.

The function that we aim to minimise, *f*(*d*), is the sum of the expected sample size of the design, evaluated at 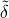, and the penalty function in equation (6).

Each iteration of the simulated annealing process consists of two steps. The first step is to generate a new candidate design from the design that the process is currently in. The second step is to decide whether the process should move from the current design to the candidate design. Both steps rely on the so-called ‘temperature’ parameters. At the end of each iteration, the temperature parameters are both reduced. We discuss both steps and the role of the temperature parameters in more detail in the following text.

We define the temperature parameters affecting the candidate design-generating process as *t*_*c*1_ and *t*_*c*2_. Then for each iteration, if the current design is *d* = (*n*_1_,*f*_1_, …, *f*_*K*−1_,*e*_1_, …, *e*_*K*_), a new candidate design *d*′ is generated by simulating a vector of 2*K*-independent normally distributed random variables, *ε*, each component of which have mean zero. The variance of the first component is *t*_*c*1_, and the variances of the others are all equal to *t*_*c*2_. The candidate design is *d*′ = *d*+ *ε*. Because *n*_1_ is generally substantially larger than the stopping boundaries, we take *t*_*c*1_ > *t*_*c*2_. Because a valid design must have *n*_1_ > 0 and *e*_*k*_ > *f*_*k*_ ∀ *k*, candidate designs are generated until one satisfies these constraints. Note that the size of *t*_*c*1_ and *t*_*c*2_ represents the degree to which the candidate design may deviate from the current design.

We define the temperature parameter representing whether the candidate design is accepted or rejected as *t*_*a*_. Let the current and candidate designs, *d* and *d*′, have function values *f*(*d*) and *f*(*d*′). Then, if *f*(*d*′) *≤ f*(*d*), the candidate design is accepted with probability 1, and if *f*(*d*′) > *f*(*d*), the candidate design is accepted with probability 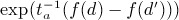. Here, the size of *t*_*a*_ governs the chance of the process allowing a move to a worse design. As *t*_*a*_ tends towards 0, candidate designs are only accepted if they improve the function value.

After each iteration, the temperatures are reduced (annealed) according to some specified function. We use 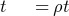, where *ρ* < 1 is a constant. We run the process for 10 000 iterations and then reset the temperatures to the starting values. We then repeat this a number of times, until no improvement in function value is seen from the previous block of 10 000 iterations. Allowing the temperatures to be reset reduces the chance of the process ending up in a local minimum instead of the global minimum.

Note from the previous text that the group size is not restricted to be an integer. Therefore, we conduct two separate simulated annealing processes. The first allows the group size as a continuous parameter. After that process is finished, the group size is rounded to the nearest integer. The second process allows only the stopping boundaries to be varied.

Because it is not the focus of this paper, we do not discuss in detail the choice of the starting temperature and annealing schedule. After trying several possible combinations, we found that suitable values are the following: *t*_*c*1_ equal to 10*%* of the required single-stage design sample size; *t*_*c*2_ = 3, *t*_*a*_ = 100, *ρ* = 0.999.

An implementation of the simulated annealing process in C is available online (http://sites.google.com/site/jmswason/supplementary-material).

### 2.6. Triangular design

Whitehead and Stratton [5] proposed the triangular design. The original formulation was for a symmetric design (*α* = *β*), but it can be extended to a nonsymmetric design, although the type I error and power are not exact in that case. The futility and efficacy thresholds form a triangular shape on the score statistic plane but can be easily transformed to the *z*-test plane. An advantage of the triangular design over the optimal and *δ*-minimax designs is that it is almost immediate to find and does not require any computationally intensive methods. Jennison and Turnbull [10] described further how to find the triangular design and its properties.

### 2.7. Admissible designs

Jung *et al.* [7] examined two-stage cancer trial designs with binary outcomes that can stop for futility only. A previous paper by Simon [17] had proposed null-optimal and minimax two-stage designs for such trials. The minimax design is the one that minimises the maximum sample size. Because there are generally many such designs, the minimax design is the one with the lowest expected sample size under the null. The authors noted that the optimal design has a relatively large maximum sample size and the minimax design has a relatively large expected sample size, and investigated other designs that balanced the two criteria.

To do this, the authors defined a loss function by using a weighted sum of the expected sample size under the null and the maximum sample size: 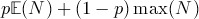, for *p* ∈ [0,1]. Admissible designs are feasible designs with 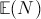 and max(*N*) such that the loss function is minimised for a particular value of *p*. The optimal and minimax designs are admissible (for *p* = 1 and 0, respectively), but other designs also exist that balance the two quantities in different ways.

For designs that balance maximum expected sample size and maximum sample size, an analogous loss function is as follows:


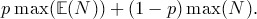
(7)

The advantage of just considering these two criteria is that it is computationally feasible to find all admissible designs when there are more than two stages. For a particular maximum sample size, the futility and efficacy parameters can be chosen so that the maximum expected sample size is minimised. Any other design with that maximum sample size cannot be admissible because Equation (7) will always be higher (unless *p* = 0).

This can be carried out for each possible maximum sample size, stopping when the *δ*-minimax design is reached. This process may be fairly computationally intensive because several simulated annealing runs must be carried out. However, after the first design is found, its futility and efficacy parameters can be used as the starting point for the next design, which reduces the amount of computation required.

### 2.8. Unknown variance

The methods in the previous sections make the assumption that *σ*^2^ is known exactly. This is not a realistic assumption, especially in early-phase trials. In this section, we propose two straightforward modifications, equivalent to approaches discussed in Jennison and Turnbull [10], that allow control of type I error when the variance is misspecified. Both methods first involve finding the stopping boundaries assuming that the variance is known and equal to *σ*^2^.

The first method is simply to estimate the standard deviation at each stage and use the *t*-test. Asymptotically, the marginal distribution of the *t*-test at each stage will converge in distribution to the *z*-test statistic when the true value of the variance is *σ*^2^. If *σ*^2^ is incorrect, using the *t*-test will better control the type I error than the *z*-test. For finite sample sizes, the type I error may still differ from the nominal level, and the power will differ from the nominal value.

A further correction to the stopping boundaries for small sample sizes can be made straightforwardly. Let *f*_*i*_ and *e*_*i*_ be the stopping boundaries for analysis *i* and *n*_*i*_ be the planned number of patients per arm to be randomised by the time of the analysis. Then, the *p*-value thresholds for stopping are given by the quantile of the normal distribution: 1 −*Φ*(*e*_*i*_) and 1 −*Φ*(*f*_*i*_), respectively. With unknown variance, when *δ* = 0, the test-statistics would be marginally distributed as a Student's *t*-distribution with 2*n*_*i*_ −2 degrees of freedom. Therefore, the new stopping boundaries 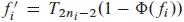 and 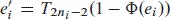, where *T*_*p*_ is the cumulative density function of Student's *t*-distribution with *p* degrees of freedom, will marginally have the correct type I error. The overall type I error of the trial may still differ from nominal because the assumed correlation between test-statistics when the variance is known will differ from the actual correlation.

## 3. Comparison of optimal and triangular designs

Using the techniques described in Section 2, we found the null-optimal, CRD-optimal, *δ*-minimax, and triangular designs for (*α*,*β*) = (0.05,0.1). We varied the number of stages, *K*, from 2 to 5. The design parameters used were *δ*_0_ = 0,*δ*_1_ = 1,*σ* = 3, for which the single-stage design requires 155 patients per arm. In the supplementary material, we show results for (*α*,*β*) = (0.05,0.2) and (0.1,0.1), and for different values of 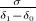. We also provide a C++ programme to allow the design to be found for the reader's choice of parameters (http://sites.google.com/site/jmswason/supplementary-material).

**Figure 1:**
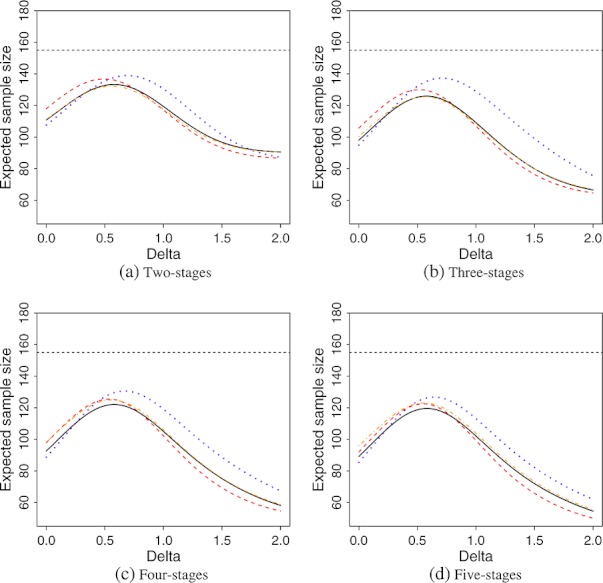
Plot of expected sample sizes per arm of the null-optimal design (dotted), the CRD-optimal design (dashed), the *δ*-minimax design (solid), and the triangular design (dash-dotted; often obscured by the solid line) for (*α*,*β*) = (0.05,0.1). The horizontal dashed line represents sample size of single-stage design.

Figure 1 shows expected sample sizes of the four designs as the true mean treatment effect, *δ*, changes. We consider values of *δ* between the null and twice the CRD, in this case, in the interval [0,2]. Table I shows the maximum sample size and the expected sample size at *δ*_0_,*δ*_1_ and 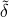 of each design.

**Table I:** Expected and maximum sample sizes per arm of investigated designs for different numbers of stages

		Null-optimal	CRD-optimal	*δ*-minimax	Triangular design
*K* = 2	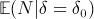	107.6	118.0	110.9	111.2
	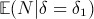	130.5	117.1	119.4	117.6
	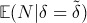	138.9	136.8	133.3	132.2
	Maximum sample size	170	172	180	180
*K* = 3	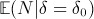	94.9	105.7	98.0	100.4
	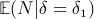	128.9	107.0	109.2	108.4
	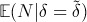	137.3	130.0	125.9	125.5
	Maximum sample size	183	186	189	192
*K* = 4	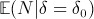	88.7	98.0	92.7	98.3
	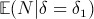	119.1	102.2	105.0	106.1
	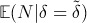	130.6	125.5	122.0	124.9
	Maximum sample size	192	196	196	204
*K* = 5	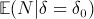	85.4	92.1	89.2	96.0
	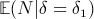	113.1	99.3	102.8	103.9
	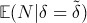	126.8	122.5	119.6	123.0
	Maximum sample size	200	210	205	210

We compare the *δ*-minimax and triangular designs. In the case of a symmetric fully sequential design, the triangular design minimises the maximum expected sample size when *α* is close to 0. Therefore, we would expect the two designs to be fairly similar in terms of expected sample size.

When *K* = 2 or 3, there is very little difference between the expected sample sizes from the designs. In fact, the *δ*-minimax design actually has a higher maximum expected sample size for *K* = 2,3. This is because the method for finding the triangular design for given *α* ≠ *β* does not result in the constraints being met exactly (the triangular design has *α* = 0.0517 and 0.0512, respectively). For *K* = 4 and 5, there is more of a difference, with the *δ*-minimax design having a 7.1 *%* reduction in expected sample size under the null and a 5.7*%* reduction for *K* = 4, compared with the triangular design.

Although the expected sample size patterns are similar, the stopping boundaries of the *δ*-minimax and triangular designs are somewhat different. Table II shows the design parameters for each five-stage design. Generally, the *δ*-minimax design is marginally more likely to stop at the first stage, although this is balanced by it being slightly less likely to stop once the trial is at a later stage. The maximum sample sizes are similar but differ between the designs for some values of *K* (Table I).

**Table II:** Parameters for five-stage designs

Design	*n*_1_	*f*_1_	*e*_1_	*f*_2_	*e*_2_	*f*_3_	*e*_3_	*f*_4_	*e*_4_	*f*_5_	*e*_5_
Null-optimal	40	−0.24	3.01	0.37	2.47	0.76	2.23	1.09	2.03	1.56	1.56
CRD-optimal	42	−0.51	2.14	0.29	2.05	0.83	2.09	1.33	2.15	2.05	2.05
*δ*-minimax	41	−0.52	2.54	0.34	2.09	0.92	2.03	1.38	1.96	1.83	1.83
Triangular	42	−0.85	2.55	0.30	2.10	0.98	1.96	1.49	1.91	1.90	1.90

Compared with the two other optimal designs, the *δ*-minimax design has desirable properties. By definition, it has the lowest maximum expected sample size of the three designs, and it also appears to do well across the range of treatment effects considered. When the treatment effect is close to *δ*_0_, the expected sample size is only slightly higher than that of the null-optimal design; when close to *δ*_1_, it is only slightly higher than that of the CRD-optimal design. The optimal designs perform well when *δ* is close to the treatment effect for which they are optimal but have considerably higher expected sample sizes when *δ* is different. As the number of stages increases, the expected sample size curves shifts downwards, indicating that including more stages results in lower expected sample sizes at each value of *δ*. The relative shapes of the curves change slightly, especially as *δ* increases past *δ*_1_.

Although minimising the expected sample size is one aspect of improving the efficiency of trials, it is also of interest to control the maximum potential sample size. If a design results in a small improvement in expected sample size at a cost of a large increase in maximum sample size, it is unlikely to be preferred in practice. Table I shows that the *δ*-minimax and triangular designs generally have a slightly larger maximum sample size for each value of *K* considered, although the difference varies with the number of stages.

Table II shows that the null-optimal design has high early futility boundaries, making stopping for futility more likely; the CRD-optimal design puts more emphasis on lowering the early efficacy boundaries; the *δ*-minimax design puts weight on both types of boundaries. These properties result in the lower expected sample sizes under the null, CRD and 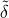, respectively.

As shown in the supplementary material, the relative performance, in terms of expected sample size, of the different designs is qualitatively similar for different values of *α* and *β* and for different values of *σ*. When *β* is increased to 0.2, the CRD-optimal and *δ*-minimax designs perform similarly in terms of expected sample size. A similar result for two-stage designs was found in Wason and Mander 4.

## 4. Unknown variance

Table III shows the actual type I error and power for the three-stage *δ*-minimax design in Table II as the true value of *σ* differs from 3. Three scenarios are considered: (i) no modification is made; (ii) *t*-tests are used; and (iii) the stopping boundaries are modified as indicated in Section 10 and *t*-tests are used. The type I error and power are estimated from 250 000 replicates each.

**Table III:** Type I error and power estimates as the true standard deviation varies from the assumed value of 3

	Type I error			Power		
*σ*	*z*-test	*t*-test	*t*-test with modified boundaries	*z*-test	*t*-test	*t*-test with modified boundaries
1	0.000	0.051	0.050	1.000	1.000	1.000
1.5	0.000	0.052	0.050	0.998	1.000	1.000
2	0.000	0.051	0.050	0.984	0.995	0.995
2.5	0.021	0.052	0.050	0.95	0.965	0.965
3	0.050	0.051	0.050	0.900	0.900	0.899
3.5	0.086	0.052	0.050	0.851	0.810	0.809
4	0.124	0.052	0.051	0.807	0.714	0.712
4.5	0.158	0.052	0.051	0.768	0.626	0.623
5	0.189	0.051	0.050	0.737	0.550	0.547

In terms of controlling the type I error, using the *t*-test in conjunction with the modified boundaries performs well. In this case, the estimated type I error is extremely close to the nominal value. The small deviations can be explained by sampling variation. Using the *t*-test without modifying the boundaries performs almost as well, with a small inflation in type I error. Using the *z*-test without taking the unknown variance into account leads to large deviations in nominal type I error.

The power results show that there is little loss from using the modified *t*-test boundaries when *σ* = 3. Although as *σ* deviates from 3, the power deviates more from nominal in comparison with assuming known variance, this is explainable by considering the type I error probability. For example, although the power of the *t*-test statistic with modified stopping boundaries is lower for *σ* = 5, this is because the type I error rate is controlled at 0.05. The *z*-test on the other hand has a large inflation in type I error, and so increased power is natural.

For this example, it appears that the assumption of known variance is not restrictive. Designing the study assuming the variance is known and then analysing it using *t*-tests results in a procedure that controls the type I error well. The power only drops if the actual variance is higher than the assumed value.

## 5. Case study—admissible designs

The *δ*-minimax design has good properties in terms of expected sample size but at a cost of somewhat higher maximum sample size compared with the null-optimal and CRD-optimal designs. The maximum sample size is certainly of interest to trial organisers and funding bodies because it represents the potential cost and recruitment time of the trial. Here, we consider designs that are admissible with respect to maximum expected sample size and maximum sample size. By studying such admissible designs, we may find ones that have good expected sample size properties without having undesirably large maximum sample sizes.

As an illustration, we consider the design of a trial that was to investigate the effectiveness of a novel drug compared with placebo in the control of diabetic neuropathic pain. Whitehead *et al.* [18] discussed design considerations. The assumed value for the standard deviation in both arms was *σ* = 2.3. The CRD was *δ*_1_ = 1. Various two-stage designs for different types I and II error probabilities and allocation ratio between the active and placebo arm were considered. The design chosen had *α* = 0.025, *β* = 0.2 and a 2:1 allocation ratio in favour of the active arm. We consider the more common situation of a 1:1 allocation ratio but keep the other parameters the same. This corresponds to design 2 in table I of [18].

**Figure 2:**
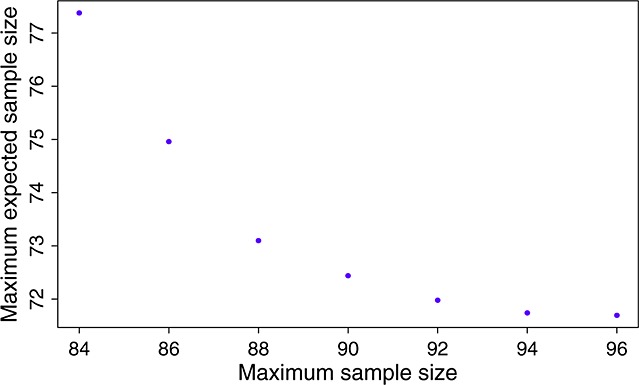
Admissible designs for different maximum sample sizes, *K* = 2,(*α*,*β*) = (0.025,0.2). Maximum sample size is per arm.

We found the admissible two-stage designs for the aforementioned parameters. For each possible maximum sample size per arm, the design with the lowest maximum expected sample size was found using simulated annealing. When the *δ*-minimax design was reached, the procedure stopped because going further would result in designs with higher maximum sample size and maximum expected sample sizes. Figure 2 shows the maximum sample size and maximum expected sample size for each found design. Note that the maximum sample size per arm is restricted to be even because there are two stages. Designs that lie on the convex hull of the set of points are admissible [19]. For two stages, all the designs found by the aforementioned process were admissible; although for three stages, one design was not (data not shown).

Figure 2 shows that for a small increase in maximum expected sample size (compared with that of the *δ*-minimax design), a sizeable reduction in maximum sample size can be made. It is still not clear which of the admissible designs is ‘best’ because each minimises the loss function for a different weighting of the maximum expected and maximum sample size. Table IV lists some factors of interest for each admissible design. It confirms that 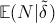 is a good surrogate for jointly considering 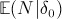 and 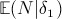 because the two latter quantities generally decrease as the former does. The only exception to this is the last design, which has a very small reduction in 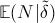 and a very small increase in 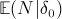 and 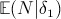 compared with the penultimate design.

The value of *p* gives the weighting of the maximum expected sample size. If *p* = 0, the only criterion of interest is the maximum sample size, whereas *p* = 1 means only the maximum expected sample size is of interest. The table includes the range of *p*'s for which each design is best. For instance, if the two quantities are each given equal weight (*p* = 0.5), the second design in the table is the best one to pick. If the trial is being carried out in an area with limited patient numbers, *p* might be chosen to be low, reflecting that the maximum sample size requires stricter control. In other situations, a higher value of *p* may be preferred because, on average, the number of patients required is reduced. However one chooses to weight the expected and maximum sample sizes, it seems unlikely that the actual *δ*-minimax design would be the most desirable one—by accepting a very small increase in expected sample size, one can get a design with a relatively large reduction in maximum sample size.

**Table IV:** Properties of admissible designs

*N* per arm	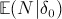	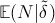	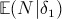	Ratio of max(*N*) to single-stage sample size	*p*-interval
84	63.41	77.38	74.23	1	[0, 0.453)
86	60.03	74.96	71.08	1.02	[0.453, 0.519)
88	56.27	73.10	70.40	1.05	[0.519, 0.752)
90	56.02	72.44	69.50	1.07	[0.752, 0.814)
92	55.63	71.98	69.18	1.10	[0.814, 0.894)
94	55.56	71.74	69.05	1.12	[0.894, 0.978)
96	55.61	71.70	69.18	1.14	[0.978, 1]

## 6. Discussion

In this paper, we have extended the investigation of the *δ*-minimax design, previously discussed by Schuster [3] for two-stage trials with binary endpoints and by Wason and Mander 4 for two-stage trials with continuous endpoints. We have examined the properties when the number of stages is increased; compared it with the triangular design, which was previously shown to minimise the maximum expected sample size when *α* tends to 0; and discussed a way of overcoming the relatively poor maximum sample size properties by using admissible designs.

In terms of expected sample size, the *δ*-minimax design has very good properties. For treatment effects close to the null, the *δ*-minimax design has an expected sample size close to that of the null-optimal design; for treatment effects close to the CRD, it has an expected sample size close to that of the CRD-optimal design. This is the case for each significance and power level considered. As the number of interim analyses increases, the patterns generally remain similar, although the whole expected sample size curve is lower. This is consistent with previous papers about optimal multistage designs 13,14,15.

*δ*-minimax designs give similar expected sample sizes to the triangular design. There is a more noticeable difference for four-stage and five-stage designs, with the *δ*-minimax design generally doing slightly better near the null. The triangular design has the advantage of being far easier to find, especially when the number of interim analyses is larger. It is almost immediate to find, whereas a reliable search for the *δ*-minimax design takes around 10 min for a five-stage design. Both designs have the undesirable property that the maximum sample size is somewhat larger in comparison with other multistage designs. This could be a problem when the maximum sample size is of particular interest, for example, when there is a limit on the number of patients that can be recruited. To overcome this drawback, designs can be found that balance the maximum sample size and the maximum expected sample size. The *δ*-minimax design in this sense is more flexible than the triangular design because the search can be restricted to certain maximum sample sizes. In the simulated annealing process, an additional penalty parameter could be applied to designs that have maximum sample sizes that are too large. There is no obvious method of generalising the triangular design so that the maximum sample size is controlled in this way.

A more principled way of approaching the problem of balancing the maximum sample size and the maximum expected sample size is using the methods discussed in Jung *et al.* [7]. In that paper, the authors considered designs that were admissible with respect to the expected sample size under the null and the maximum sample size. When stopping for efficacy is permitted, it is wise to consider the expected sample size for treatment effects away from the null too. One method is to include the expected sample size under the CRD as a third criterion. This would make things computationally difficult because all feasible designs must be searched over to check for admissibility. For a two-stage design, this is possible, but it becomes impractical to search all designs when *K* > 2. Instead, we suggest using the maximum expected sample size as a surrogate for the entire expected sample size curve because designs that have low maximum expected sample sizes generally do well under the null and CRD values too.

Examining the range of admissible designs shows that substantial savings in maximum sample size can be made with only a tiny increase in maximum expected sample size. For the case study, a design existed with a 4.2 *%* drop in maximum sample size at the cost of a 0.4 *%* increase in maximum expected sample size. It certainly seems that some admissible designs have very appealing properties and are highly recommended for use in real trials. Before choosing an admissible design, thought must be put into choosing how the maximum sample size and maximum expected sample size are weighted. This will likely reflect the potential number of patients that can be recruited and the cost per patient.

One admissible design is the ‘minimax design’, which has the lowest maximum sample size. In [17], the author more thoroughly considered the minimax design. When stopping for efficacy is not allowed, the minimax design is one with the minimum possible maximum sample size. Because many such designs may exist, the expected sample size under the null is used as a secondary criterion. In the case of stopping for efficacy being allowed, the minimax design becomes less relevant because it is not obvious what to use as a secondary criterion. In [20], Mander and Thompson considered two minimax designs, which had the expected sample sizes under the null and alternative hypotheses as secondary criteria, respectively. The minimax design here is a third type—using the maximum expected sample size as a secondary criterion.

In this paper, we assumed that the standard deviation, *σ*, of the outcome is known. We also proposed two straightforward procedures to control the type I error. The first is to design the trial assuming known variance and then use *t*-tests in the analysis. The second is to modify the stopping boundaries by finding the equivalent *p*-values of each stopping boundary and then using the relevant quantile from the *t*-distribution instead. For the relatively large sample sizes considered, the second approach controls the type I error at the nominal level. For smaller trials, it still performs well, although shows a small inflation in type I error (data not shown).

Although the type I error can be controlled at the correct level, deviations from the planned value of *σ* strongly affect the power. If the design is fixed in advance, then there is no way to overcome this. An alternative class of design to use is one in which the sample size is re-estimated at each stage. Proschan [21] provided a recent discussion of this concept. Sample size re-estimation allows the power to be controlled when *σ* differs from the planned value, for example, when little information is available about the value of *σ* before the trial. In practice, some constraints are required for such a design. For example, if the apparent standard deviation is above some threshold, then the trial may have to be stopped for futility. This would allow a maximum sample size to be known in advance, which is desirable for planning purposes. Considering designing optimal trials allowing for sample-size re-estimation would be an interesting piece of future work.

The ideal solution for unknown *σ* would be to derive the joint distribution of *t*-tests, which use all patients randomised by the time of the analysis. Unfortunately, finding the joint distribution of such *t*-tests is less straightforward than for *z*-tests. Exact derivations require integration over more variables 22 and so are time consuming in comparison with the methods examined in this paper. Simulation is possible [23] but is even more time consuming. In a study by Wason and Mander 4, the first stage test statistic was the *t*-test statistic from the first *n*_1_ patients and the second stage test statistic was a weighted average of the first-stage and second-stage *t*-test statistics. This could be extended to multistage designs but has the disadvantage of losing information about the standard deviation estimate from previous stages. If the sample size is large, then this should not make much difference to the trial properties. In smaller trials, where the standard deviation will be estimated imprecisely, it could affect the sample size required more severely. Approximate methods exist, such as the quantile-replacement method discussed by Pocock [1], which are quick and produce designs with reasonable accuracy in terms of significance level. In addition, the trial can be designed as if the variance is known, with the resulting stopping boundaries being used for *t*-tests instead. This leads to inflated errors when the trial has a low group size [23] but is adequate otherwise. Work that reviews the different approaches in the context of optimal designs would be very useful.

Simulated annealing as a method for finding *δ*-minimax designs works very well for designs with a low number of stages. When the number of stages increases, the method becomes less consistent, and several independent runs must be carried out to be sure of finding the correct design. Lai [6] considered a sequential symmetric design (i.e. an interim analysis is carried out after each response is observed and *α* = *β*), which minimises the maximum expected sample size. It is possible that this method can be extended to nonsymmetric group-sequential designs, as it was for the triangular design. Doing this would allow designs with larger numbers of stages to be found more quickly and reliably. However, it is anticipated that going beyond *K* = 5 would have limited gains and would involve designs that were difficult to implement in practice.

Overall, we have shown in this paper that designs that focus on the maximum expected sample size have good expected sample size properties in comparison with other optimal designs, are readily communicated and can be easily adapted to have good maximum sample size properties. The use of such designs could help improve the efficiency of the drug development process.
